# Assessment of Chemical Composition and In Vitro Antioxidant, Antidiabetic, Anticholinesterase and Microbial Virulence-Quenching Effects of Salad Burnet (*Sanguisorba minor* L.) Harvested from Algeria

**DOI:** 10.3390/plants12244134

**Published:** 2023-12-11

**Authors:** Chahrazed Haouam, Sameh Boudiba, Alfred Ngenge Tamfu, Selcuk Kucukaydin, Karima Hanini, Haouaouchi Fatma Zohra, Soraya Hioun, Andreea Dediu Botezatu, Özgür Ceylan, Louiza Boudiba, Mehmet Emin Duru, Rodica Mihaela Dinica

**Affiliations:** 1 Laboratory of Applied Chemistry and Renewable Energies (LACRE), Echahid Cheikh Larbi Tebessi University, Constantine Road, Tebessa 12002, Algeriafatmazohra.haouaouchi@univ-tebessa.dz (H.F.Z.);; 2 Department of Chemical Engineering, School of Chemical Engineering and Mineral Industries, University of Ngaoundere, Ngaoundere 454, Cameroon; 3 Food Quality Control and Analysis Program, Ula Ali Kocman Vocational School, Mugla Sitki Kocman University, Mugla 48147, Turkey; 4 Department of Chemistry, Faculty of Science, Mugla Sitki Kocman University, Mugla 48000, Turkey; 5 Department of Medical Services and Techniques, Koycegiz Vocational School of Health Services, Mugla Sitki Kocman University, Mugla 48800, Turkey; 6 Laboratory of Organic Materials and Heterochemistry (LOMH), Echahid Cheikh Larbi Tebessi University, Constantine Road, Tebessa 12002, Algeria; 7 Department of Natural and Life Sciences FSESNV, Echahid Cheikh Larbi Tebessi University, Constantine Road, Tebessa 12002, Algeria; 8 Department of Chemistry, Physics and Environment, Faculty of Sciences and Environment, Dunarea de Jos University, 47 Domneasca Str., 800008 Galati, Romania

**Keywords:** *Sanguisorba minor*, chemical composition, antioxidant activity, antibiofilm properties, anticholinesterase activity, antidiabetic potential

## Abstract

*Sanguisorba minor* is a medicinal vegetable used in seasoning desserts, juices, and beverages. An evaluation of the total flavonoid, phenolic, tannin and anthocyanin contents indicated that these classes of compounds are distributed variably in the different fractions. In summary, the HPLC-DAD analyses enabled the identification and quantification of thirteen phenolic compounds in an ethyl acetate extract (EAE), nine in a dichloromethane extract (DCME), seven in an aqueous extract (AQE) and four in a butanol extract (BE). Rutin was the most abundant phenolic compound in the BE (278.4 ± 1.20 µg/g) and AQE (32.87 ± 0.23 µg/g) fractions, while apigenin was the most abundant in the DCME (84.75 ± 0.60 µg/g) and EAE (156.8 ± 0.95 µg/g) fractions. The presence of phenolic compounds in the fractions conferred good antioxidant capacity, especially the EAE and DCME fractions, which both exhibited higher antioxidant effects than BHA and α-tocopherol in DPPH^•^ and CUPRAC assays. Additionally, in the ABTS^•+^ assay, EAE (IC_50_ = 9.27 ± 0.33 µg/mL) was more active than α-tocopherol (IC_50_ = 35.50 ± 0.55 µg/mL), and BHA (IC_50_ = 12.70 ± 0.10 µg/mL). At 200 µg/mL, the fractions inhibited acetylcholinesterase and butyrylcholinesterase as well as α-amylase and α-glucosidase, indicating that they can slow neurodegeneration and hyperglycemia. Minimal inhibitory concentration (MIC) values ranged from 0.312 mg/mL to 1.25 mg/mL, and fractions showed good biofilm inhibition against *Staphylococcus aureus* and *Escherichia coli*. The extracts exhibited good violacein inhibition in *Chromobacterium violaceum* CV12472 and *Chromobacterium violaceum* CV026, despite the supply of external acyl-homoserine lactone to CV026. The antioxidant, quorum-sensing, antibiofilm and enzyme inhibition attributes indicate the potential for the application of *S. minor* as a food preservative.

## 1. Introduction

The growing resistance to antibiotics of pathogenic bacteria constitutes a challenging problem for the food and drug industries when bacteria mutate under adverse conditions and become unsusceptible to the available drugs [1]. Pathogenic bacteria and fungi cause foodborne diseases, which expose many people to risk, especially young children, pregnant women, old people, and those with weak immune systems [2]. Antibiotics that target bacterial growth inhibition or death are gradually falling out of use due to resistance resulting from biofilm colonies [3]. Biofilm colonies account for surface contamination, nosocomial infections, fatal food poisoning, food decay and many other health threats. The ineffectiveness of antibiotics is due to the growth of multidrug resistance (MDR) [4,5] resulting from various virulence factors such as bacterial biofilms [6], swarming motility [7] and cellular quorum-sensing (QS) activities [8]. A novel biocontrol strategy to overcome bacterial resistance can be achieved through the inhibition of the expression of virulence factors through quorum-sensing (QS)-mediated processes in pathogenic microorganisms [8]. QS involves a cell-to-cell communication network that enables the bacterial colonies to monitor their milieu and control production of signal molecules such as violacein production, toxin production, swarming motility, and biofilm formation [9]. *S. minor* has been described as possessing antibacterial, antifungal and antiviral activities [10] but the effects of the plant on virulence factors such as biofilm, swarming and QS inhibition are inexistent.

Enzymes are involved in most biochemical processes in the living system, and an imbalance of enzyme activity can lead to pathological conditions such as inflammation, microbial infections, diabetes, HIV, Alzheimer’s disease (AD), and hyperpigmentation [11,12,13]. Correcting metabolic imbalances can be provided by blocking enzyme activity using inhibitors which can be reversible or irreversible. Alzheimer’s disease causes cognitive decline, loss of memory, and death in old people, and is one of the significant causes of acetylcholine deficiency. The breakdown of acetylcholine, which is an important neurotransmitter that interferes in the central and peripheral nervous systems, could lead to serious complications such as Alzheimer’s disease, and substances that can inhibit cholinesterases are employed as a remedy [14,15]. Diabetes is a deadly metabolic disease, and solutions such as diet control, physical exercise, and hypoglycemic drugs are used, but most of these drugs can produce undesirable side effects [16]. Two key enzymes, α-glycosidase, and α-amylase are responsible for carbohydrate breakdown into sugars, especially glucose, and the inhibition of these two enzymes can slow the conversion of carbohydrates into glucose and lower blood glucose levels; hence, a suitable strategy to remedy type 2 diabetes [14,17,18]. *S. minor* has significant antioxidant and neuroprotective activities which could be attributed to the rich polyphenol profile of the plant’s extracts, thereby making the plant important in overcoming oxidative damage [10]. *S. minor* protects the brain from damage and equally inhibits some oxidative enzymes such as superoxide dismutase (SOD) and catalase (CAT) as well as cholinesterases [19].

*Sanguisorba minor* L., also called salad burnet, is an edible perennial herb with pinnate leaves and red-green petals [20]. *S. minor* is a herbaceous perennial plant in the Rosaceae family widely distributed across Europe, Asia, and northern Africa and commonly found in the dry and semi-dry grasslands of the Mediterranean regions. This plant is a food additive to fruit juices, cheese, ice drinks, butter, vinegar, salad, eggs, and meat dishes, as well as in the seasoning of various traditional dishes [21,22,23]. Besides its nutritional uses, this plant is also used for pharmacological purposes, which are attributable to the chemical compounds it contains. *Sanguisorba minor* L. has anti-Alzheimer [24,25,26,27], antioxidant [28], and antiviral [29,30] activities. In addition, it is used for the treatment of conjunctivitis, fever, diarrhea [21,31], intestinal infections, duodenal ulcers, bleeding, and burns [32]. *S. minor* has been shown to contain 2,3-Hexahydroxydiphenoyl-glucose, sanguiin (H-1 and H-10 derivatives), punicalagin gallate, galoyl-bis-hexahydroxydiphenyl glucoside (isomers 1 and 2), ellagic acid hexoside, ellagic acid pentoside, pedunculagin, lambertianin C, C-type (epi)catechin trimer, cyanidin-glucoside, B-type (epi)catechin dimer (isomer 1 and 2), catechin, cyanidin-malonylglucoside, quercetin-galloyl-glucoside, quercetin-glucuronide, quercetin-glucoside, quercetin-galloylhexoside, quercetin-O-hexoside gallate (isomer 1 and 2), quercetin-O-pentoside, kaempferol-glucuronide, kaempferol-3-O-glucoside, kaempferol-O-hexoside, apigenin-O-deoxyhexoside, 3-Caffeoylquinic acid (Neochlorogenic acid), caffeic acid-glucoside, 5-Caffeoylquinic acid (Chlorogenic acid), p-Coumaroylquinic acid, gallic acid glucoside, caffeoyl ester (isomer 1 and 2), digalloyl glucoside and ellagic acid, which are the major polyphenolic compounds in this species [21,33,34,35,36,37,38].

It is important to develop nutraceuticals from edible medicinal plants such as *S. minor,* by using different solvent extractions and investigating its biological activities, notably the antibiofilm, antiswarming and antiquorum-sensing effects which have not be investigated previously. In this study, extracts of *S. minor* were prepared, and their phenolic profiles were established using HPLC-DAD. Their antidiabetic, anticholinesterase, antioxidant, antiquorum-sensing, antiswarming and antibiofilm activities were investigated and reported.

## 2. Results

### 2.1. Yields of Obtained Extracts

**Figure 1:**
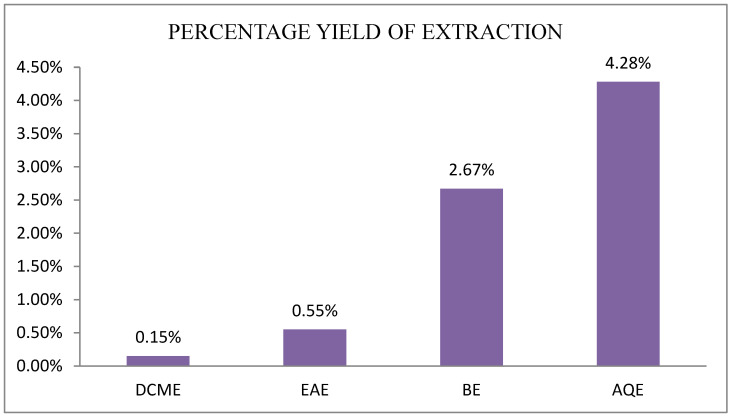
Histogram of extraction yields of *Sanguisorba minor*.

Polar solvents are favorable in the extraction of maximum amounts of phenolic compounds; therefore, methanol was used to obtain a crude fraction, followed by liquid–liquid extraction with other solvents to give fractions with different yields. Fractions from the four solvents: dichloromethane (DCME), ethyl acetate (EAE), n-butanol (BE), and distilled water (AQE) were obtained from the crude extract of *S. minor*. The percentage yields are presented in Figure 1, and the highest ones were observed for the aqueous extract at 4.28%, followed by the n-butanol at 2.67%. The yields show that the plant compounds have different affinities with the solvents, and the polar solvents extracted more phytoconstituents than the less polar ones.

### 2.2. Total Polyphenols, Flavonoids, Tannins, and Anthocyanin Contents

The total phenolic content (TPC), total flavonoid content (TFC), condensed tannin content (TC) as well as the anthocyanin contents (AC) of the different fractions of *S. minor* are reported in Table 1. The TPC is expressed in terms of milligrams of gallic acid equivalents per gram of dry weight of extract (mg GAE/g DW), TFC is expressed in terms of milligrams of quercetin equivalents per gram of dry weight of extract (mg QE/g DW), TC is expressed in terms of milligrams of catechin equivalents per gram of dry weight of extract (mg CE/g DW) while the AC is expressed in terms of milligrams of Cyanidin-3-galactoside equivalents per gram of dry weight of extract (mg CGE/g DW). From the results of the analysis of TPC, TFC, TC, and AC, it was observed that these classes of compounds were distributed variably in the different fractions and this was due to the nature of the solvents’ polarity. For the total phenolic content, the EAE (811.0 ± 4.7 mg GAE/g DW) contained the highest amount of phenolic compounds, followed by BE (461.0 ± 4.8 mg GAE/g DW), while DCME (240.0 ± 4.9 mg GAE/g DW) had the smallest amount of phenolics. For the total flavonoid contents, the EAE (254.9 ± 3.5 mg QE/g DW) contained notably the highest amounts, while DCME (155.7 ± 1.1 mg QE/g DW) and BE (101.6 ± 2.0 mg QE/g DW) had average amounts of flavonoids and the smallest amount was found in the AQE (78.4 ± 2.2 mg QE/g DW). Concerning the tannin contents, the different fractions in descending amounts of their TC were revealed as EAE (129.7 ± 2.1 mg CE/g DW), DCME (113.5 ± 3.0 mg CE/g DW), BE (102.2 ± 4.3 mg CE/g DW) and AQE (58.6 ±1.6 mg CE/g DW). The anthocyanin contents indicated that they were a minor class of metabolites as the AC varied from 8.6 ± 0.2 mg CGE/g DW in the BE to 1.5 ± 0.0 mg CGE/g DW in AQE.

### 2.3. Identification and Quantification of Phenolic Compounds by HPLC-DAD

**Figure 2:**
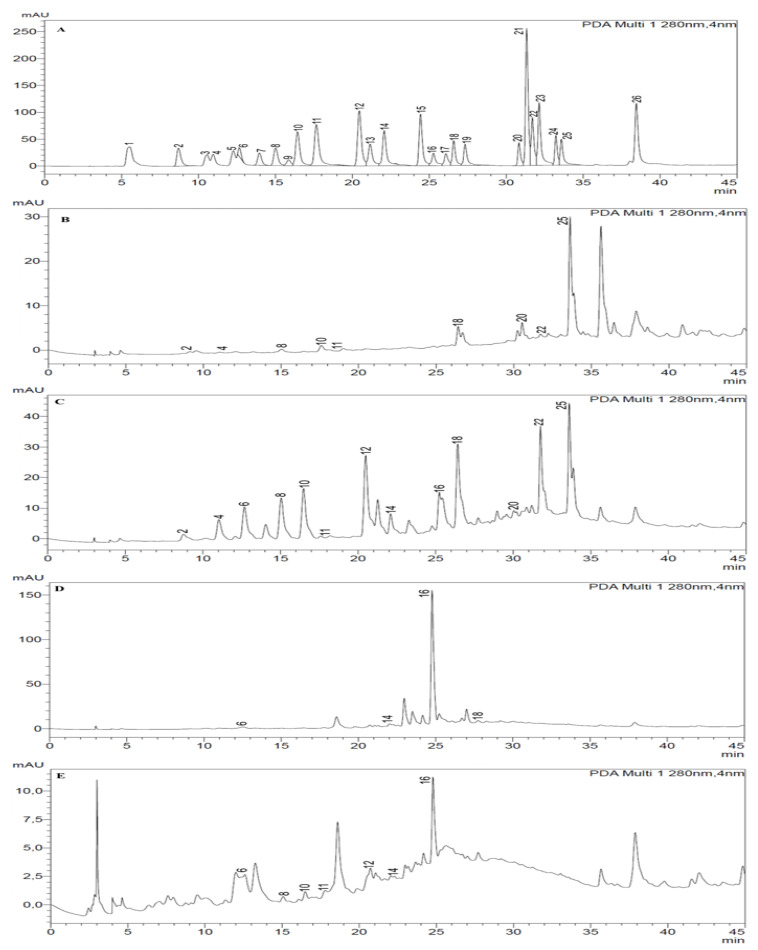
HPLC chromatograms of phenolic compounds: (**A**) standards, (**B**) DCME, (**C**) EAE, (**D**) BE and (**E**) AQE fractions of *Sanguisorba minor*.

**Figure 3:**
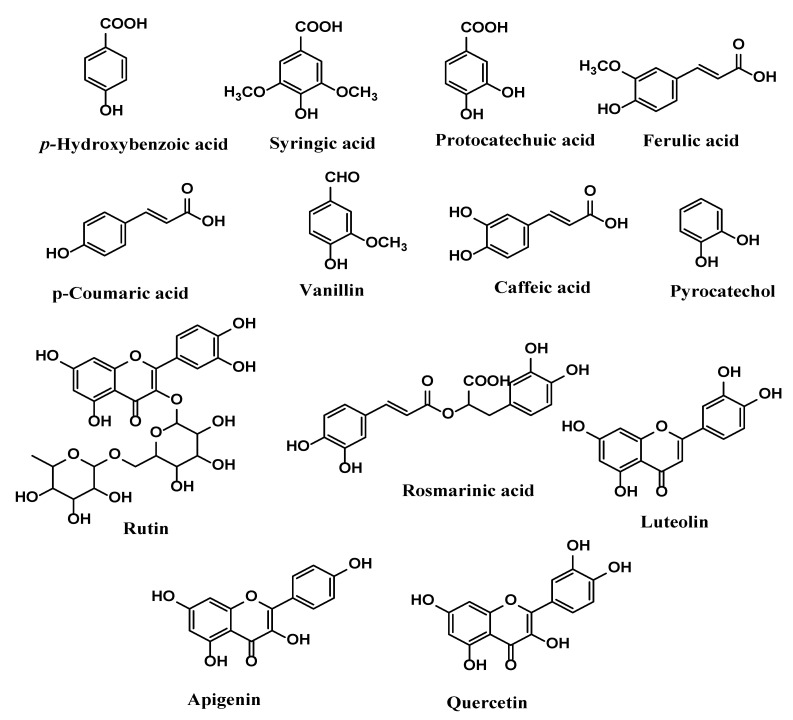
Structures of phenolic compounds identified in fractions of *Sanguisorba minor*.

Thirteen phenolic compounds were detected and quantified in the different fractions of *S. minor*, confirming that the distribution between the solvents of each detected phenolic compound is affected by the extraction process since the quantity varied from one solvent to another. The HPLC chromatograms are provided in Figure 2. The twenty-six standard phenolic compounds used for comparison with those in each fraction and the identified ones are presented in Table 2. In summary, the phenolic compounds identified and quantified in each fraction were thirteen in the EAE, nine in the DCME, seven in the AQE and four in the BE fraction. Rutin was the most abundant compound in the AQE (32.87 ± 0.23 µg/g) and BE (278.4 ± 1.20 µg/g) fractions, while apigenin was the most abundant compound in the EAE (156.8 ± 0.95 µg/g) and DCME (84.75 ± 0.60 µg/g). It is worth highlighting that all the identified compounds were present in the EAE in different proportions and the other significant amounts of detected compounds in the EAE were as follows: luteolin (133.6 ± 0.70 µg/g), rosmarinic acid (124.5 ± 0.80 µg/g), *p*-coumaric acid (120.1 ± 0.75 µg/g), caffeic acid (70.49 ± 0.35 µg/g), and syringic acid (75.82 ± 0.48 µg/g). The structures of the identified phenolic compounds are given in Figure 3.

### 2.4. Antioxidant Activity

The antioxidant activity evaluated through six different assays is reported in Table 3. The EAE was the most active fraction in the β-carotene-linoleic acid (IC_50_ = 7.44 ± 0.28 µg/mL), ABTS^•+^ (IC_50_ = 9.27 ± 0.33 µg/mL), and H_2_O_2_ (IC_50_ = 49.2 ± 0.21 µg/mL) assays, while the DCME fraction had the best activity in the DPPH^•^ (IC_50_ = 7.16 ± 0.80 µg/mL), CUPRAC (IC_50_ = 8.36 ± 0.25 µg/mL), and metal chelating (IC_50_ = 5.83 ± 1.20 µg/mL) assays compared to other fractions of *S. minor*. Interestingly, these results showed that the EAE and DCME were more powerful antioxidants than the standards BHA and α-tocopherol in the DPPH^•^ and CUPRAC assays. Additionally, in the ABTS^•+^ assay, EAE (IC_50_ = 9.27 ± 0.33 µg/mL) was more active than α-tocopherol (IC_50_ = 35.50 ± 0.55 µg/mL) and BHA (IC_50_ = 12.70 ± 0.10 µg/mL). The other extracts, however, exhibited moderate antioxidant activities.

### 2.5. Anticholinesterase and Antidiabetic Activities of the S. minor

The inhibition of cholinergic enzymes AChE and BChE are beneficial health solutions for AD, and for this reason, the inhibitory activities of *S. minor* fractions were evaluated on both enzymes and are reported in Table 4. The percentage inhibitions at the highest test concentration of 200 µg/mL in the AChE inhibitory assay indicated that the EAE (36.11 ± 0.51%) and DCME (32.75 ± 0.48%) were the most active extracts as compared to the standard galantamine (88.70 ± 0.50%). The extracts showed better activity against BChE than AChE, with the most active ones being EAE (54.50 ± 0.74%) and DCME (41.25 ± 0.69%) compared to galantamine (80.20 ± 0.30%). It should be noted that the AQE was the least active against both cholinesterases. The ability of the fractions of *S. minor* to reduce carbohydrate hydrolyses by inhibiting both α-amylase and α-glucosidase is presented in Table 4. This shows that the fractions can reduce blood glucose levels by reducing the activity of these enzymes; therefore, it is an exhibition of their antidiabetic potential. The most active extract was DCME with a percentage inhibition at 200 µg/mL of 55.18 ± 0.26% and 32.45 ± 0.43% against α-glucosidase, and α-amylase, respectively. Acarbose was used as standard and showed percentage inhibitions at 200 µg/mL of 86.51 ± 0.45% and 81.33 ± 0.90% against α-glucosidase and α-amylase, respectively. The percentage inhibitions of EAE and BE were relatively moderate, while those of AQE were low. Acetylcholine is important in neurotransmission, and the effects of two cholinesterases, namely, acetylcholinesterase (AChE) and butyrylcholinesterase (BChE) usually reduce its activity.

### 2.6. Violacein and Quorum-Sensing (QS) Inhibitions

Chromobacterium species produce a purple pigment called violacein to protect their membranes against external oxidation. *C. violaceum* CV12472 produces this purple pigment violacein during its growth, while *C. violaceum* produces violacein only when an external source of acyl homoserine lactone (AHL) is provided and both cases occur through a mediated quorum-sensing process. The bacterial strain *C. violaceum* CV12472 is typically used in the qualitative screening for violacein-production inhibition, which is revealed by the measurable absence or attenuation of the violet color, conveniently measured at MIC and sub-MIC concentrations. The MIC values of the fractions as the violacein inhibition percentages are recorded in Table 5. The MIC values against *C. violaceum* CV12472 were 0.652 mg/mL for DCME and EAE, 2.5 mg/mL for BE and 1.25 mg/mL for AQE. All test samples exhibited excellent violacein inhibition with 100% inhibition percentages at MIC, and all inhibited violacein even at MIC/4. Only the fractions DCME (31.0 ± 0.7%), EAE (13.0 ± 0.5%), and AQE (24.9 ± 0.5%) showed violacein inhibition at MIC/8 inhibition. At MIC/16, only DCME and AQE showed violacein inhibition, and no sample can inhibit violacein beyond this concentration. The diameters of the QS inhibition zones of the *S. minor* fractions against *C. violaceum* CV026, under a supply of AHL, were measured at MIC and sub-MIC concentrations and are presented in Table 6. MIC values against *C. violaceum* CV026 were 1.25 mg/mL for DCME and EAE and 0.625 for BE and AQE. At MIC, DCME (14.0 ± 1.5 mm), EAE (13.0 ± 0.8 mm), BE (14.5 ± 0.6 mm) and AQE (17.0 ± 1.2 mm) all had very good quorum-sensing inhibitions (QSI). All of the fractions showed very good QSI at MIC and MIC/2. Only the most active extract, AQE, showed a QSI of 11.5 ± 0.4 mm and 09.0 ± 0.5 mm at MIC/4 and MIC/8.

### 2.7. Swarming Motility Inhibition on P. aeruginosa PA01

*P. aeruginosa* PA01 is an opportunistic flagellated bacteria commonly used as a model organism for evaluating swarming motility inhibition in microorganisms. The inhibition of the swarming movement of the fractions of *S. minor* was determined at three concentrations of 50 µg/mL, 75 µg/mL, and 100 µg/mL, and the results are presented in Table 7. The most active fractions were BE and EAE with percentage inhibitions of 39.5 ± 0.3% and 34.9 ± 1.5% at 100 µg/mL and 10.5 ± 0.3% and 06.2 ± 0.1% at 50 µg/mL, respectively. Swarming motility inhibition varied from 18.4 ± 0.6% at 100 µg/mL to 05.5 ± 0.2% at 75 µg/mL for DCME and from 20.1 ± 0.5% at 100 µg/mL to 03.8 ± 0.1% at 75 µg/mL for AQE.

### 2.8. Antimicrobial and Antibiofilm Properties

The broth microdilution method was used to determine the antimicrobial activities, and the results were recorded as the minimum inhibitory concentration (MIC) of the various fractions of *S. minor* against Gram-positive bacterium (*Staphylococcus aureus*), Gram-negative bacterium (*Escherichia coli*), and one fungus (*Candida albicans*). The fractions exhibited antibacterial and antifungal activities against the test microorganisms, and the MIC values are reported in Table 8.

The DCME had MIC values of 1.25 mg/mL against *S. aureus* and *E. coli* and 0.625 mg/mL against *C. albicans*. The EAE exhibited an MIC of 1.25 mg/mL against *S. aureus* and *C. albicans* and 2.5 mg/mL against *E. coli*. The BE had an MIC of 0.625 against *S. aureus* and *C. albicans* and an MIC of 2.5 mg/mL against *E. coli*. The most active fraction was the AQE with an MIC of 0.312 against *S. aureus*, 1.25 against *E. coli* and 0.625 mg/mL against *C. albicans*. The percentage inhibitions of the fractions were subsequently determined at MIC and sub-MIC concentrations using the crystal-staining method, and the results are shown in Table 8. The percentage inhibition of biofilms varied from 71.25 ± 1.78% (MIC) to 16.92 ± 0.56% (MIC/4) for DCME against *S. aureus.* For the same *S. aureus*, antibiofilm varied from 55.65 ± 0.59% (MIC) to 5.87 ± 0.18% (MIC/4) for EAE and from 76.14 ± 1.95% (MIC) to 10.22 ± 0.15% (MIC/8) for the most active extract, BE. The AQE had lowest antibiofilm activity against *S. aureus* and only inhibited biofilm at MIC (26.36 ± 0.27%) and MIC/2 (8.29 ± 0.06%). Against the Gram-negative bacterium *E. coli*, the antibiofilm activity varied from 60.16 ± 1.24% (MIC) to 13.15 ± 0.28% (MIC/4) for DCME and from 46.13 ± 0.78% (MIC) to 6.18 ± 0.10% (MIC/4) for BE. The biofilm percentage inhibitions ranged from 56.65 ± 1.05% (MIC) to 06.21 ± 0.32% (MIC/8) for the most active extract, EAE, and from 51.11 ± 0.64% (MIC) to 02.44 ± 0.05% (MIC/8) for AQE. Only the AQE inhibited biofilm formation in the fungus *C. albicans* with an antibiofilm activity of 11.26 ± 0.13% (MIC) and 02.44 ± 0.05% (MIC/2). *S. aureus* and *E. coli* biofilms were more susceptible to the *S. minor* fractions than *C. albicans* biofilm.

## 3. Discussion

Different structural phenolic compounds such as lignans, phenolic acids, stilbenes, and various flavonoids, are produced in plants and have considerable functions in health and foods, such as protecting the tissues from oxidative stress [39]. This phenomenon has attracted much attention from researchers to search for phenolic extracts and evaluate their bioactivities. The plant *S. minor* usually contains phenolic compounds whose amounts vary according to the regions where they are planted, the environment and harvest season [36]. Phenolic compounds are widely distributed in various food plants, spices, beverages, and many other vegetable diet items, and their consumption can protect the body from various diseases [40,41,42]. After extraction, the identification and quantification of phenolic compounds in food samples and medicinal plants can be suitably conducted by HPLC analysis because of their large amounts and structural similarities as well as close polarities, which makes their separation and identification difficult by other means [43]. Polyphenols play protective roles against UV light, pests and other stresses and provide attractive aromas and colors to insects, facts which have motivated much research concerning their extractive and analytical aspects as well as their biological activities [44,45]. Due to the importance of phenolic compounds in foods and health, *S. minor* was investigated for its phenolic composition using maceration followed by liquid–liquid extraction and characterization by HPLC-DAD. The suitable methods for the extraction of polyphenols also include liquid–liquid extraction, usually affected by solvent polarities, and the identification and quantification of phenolic compounds can equally be carried out using HPLC-DAD [46,47]. The phenolic composition of *S. minor* was reported to be rich in bioactive phenolic compounds, notably caffeic acid, syringic acid, *p*-coumaric acid, rutin, rosmarinic acid, luteolin and apigenin, which were identified and quantified in high amounts in the fractions. In one study, *S. minor* was shown to contain twenty-three compounds comprising phenolic acids, tannins and flavonoids which were identified using HPLC-DAD-MS [36]. Glycosides of quercetin, kaempferol, caffeic acid, apigenin, chlorogenic acid and chicoric acid have been described as common phenolic compounds in *S. minor* using the HPLC method [48]. In this study, similar compounds were identified but their glycosides were not detected. The differences in the chemical compositions may be explained by the differences in the geo-climatic conditions of the areas of collection and the experimental conditions. The results indicate that the method enabled the appreciable extraction of important phenolic compounds, which were distributed within the various extracts according to solvent polarities. These results are logical since Sanguisorba species are reported to contain over 270 compounds, amongst which are phenolic compounds, flavonoids, neolignans, together with triterpenoids, sterols and fatty acids, and these compounds confer numerous biological activities [24,49]. The phenolic compounds identified in *S. minor* as well as other constituents confer, probably in a synergistic manner, various biological activities including antioxidant and antimicrobial activities [50].

The antioxidant potential of *S. minor* extracts investigated through in vivo assays and different in vitro assays, including ABTS, carotene-linoleic acid assay, DPPH, CUPRAC and FRAP, has been reported [50,51] and this plant exhibits antioxidant activity in conformity with our results. Phenolic compounds are significant antioxidants in food, and they can quench free radicals and other oxidative species by electron or hydrogen transfer, loss of proton, as their chelation mechanism [52]. However, synthetic antioxidants usually show limitations, and therefore natural antioxidants from edible plants are more successful and are increasingly interesting as indispensable food additives which are capable of retarding toxic oxidation products and rancidity, while increasing the shelf-life of foods without reducing their nutritional qualities [44,53]. In this study, the various fractions of *S. minor* exhibited good antioxidant activities. In the β-carotene, ABTS^•+^, and H_2_O_2_ assays, the EAE fraction was the most active of the extracts because of the high number of identified phenolics compounds with high amounts of *p*-coumaric acid, rosmarinic acid, luteolin, and apigenin, which are antioxidant compounds. In the DPPH^•^, CUPRAC, and metal chelating assays, the DCME was the most active fraction, which could be explained by the fact that it had the highest amount of vanillin. In some other studies, methanol extracts were more active than the DCM extracts in five different assays, suggesting a higher antioxidant capacity for polar extracts than non-polar ones [51]. *S. minor* has been shown to possess strong antioxidant activity and could prevent oxidative stress diseases because of its phenolic contents [54]. In another study, *S. minor* was used to enhance the oxidative stability of edible oils [34]. The rich phenolic ingredients of *S. minor* and their significant antioxidant activity indicate their potential for application in food as natural supplements that could replace synthesized ones [24].

Besides antioxidant effects, *S. minor* was shown to possess anticholinesterase activity in the AChE inhibitory assay [34]. Inhibiting cholinesterases is a suitable solution in the treatment of Alzheimer’s disease, and there is growing interest in focusing on natural anticholinesterase compounds since synthetic ones are costly and may show side effects [46]. The fractions of *S. minor* were able to inhibit both AChE and BChE, and the EAE fraction was the most active. This fraction contained the highest number of phenolic compounds, and phenolic compounds have been described among the naturally potent anticholinesterase compounds, and foods that are rich in polyphenols can reduce the risk of Alzheimer’s disease and other non-communicable diseases [16,55,56,57]. The various dietary polyphenols including ellagic acid, rosmarinic acid, and cinnamic aldehyde possess pro-cognitive and neuroprotective potentials, which are exhibited through different modulations of pro-oxidants and other mechanisms such as the inhibition of amyloid-beta aggregation [57,58]. Besides the inhibition of cholinesterase by phenolic compounds, which provides a remedy for Alzheimer’s disease, they equally inhibit the carbohydrate hydrolyzing enzyme, which is a suitable strategy to combat type 2 diabetes mellitus and hyperglycemia, and reduce blood glucose levels [59]. The fractions of *S. minor* inhibited both α-glucosidase and α-amylase; these enzymes break down starch into simple sugars, which are absorbed into the blood, causing increased blood sugar levels and diabetes. The DCME was the most active fraction. The antidiabetic potential of *S. minor* fractions may be conferred by the phenolic compounds and other constituents that they contain. Some synthetic drugs, such as acarbose, are powerful inhibitors of starch hydrolysis, but natural enzyme inhibitors, such as food phenolics, are potentially safer for controlling hyperglycemia [60].

**Figure 4:**
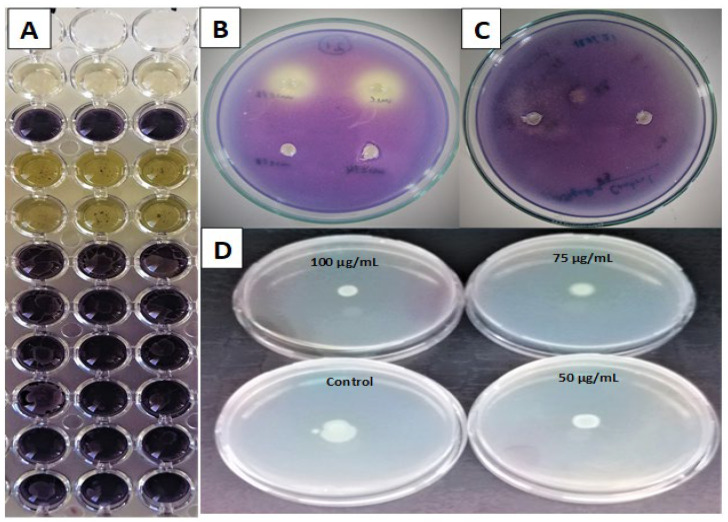
(**A**) Violacein inhibition plates against *C. violaceum* CV12472; (**B**) quorum-sensing inhibition plate against *C. violaceum* CV026; (**C**) quorum-sensing inhibition control plate; (**D**) swarming motility inhibition plates against *P. aeruginosa* PA01.

Phenolic compounds in various parts of *S. minor* are believed to confer antimicrobial effects because the parts that possess the highest phenolics showed the highest antibacterial activities [35]. However, the effects of *S. minor* on virulent factors of bacteria such as biofilm formation, quorum-sensing and swarming motilities have not been reported. Many virulent processes involved in microbial pathogenicity, such as biofilm formation, sporulation, toxin production, pigment formation, swarming motility and enzyme secretion are mediated through quorum-sensing and the inhibition of QS is an opportunity to combat pathogens since QS helps bacteria to develop resistance and evade antibiotic effects [61,62,63,64]. Production of violacein by chromobacterium represents a QS process and it is easily measurable and can represent both signal reception as well as signal production [65]. This is indicated by a decrease in the violacein pigment that varies with concentration as shown in Figure 4. Since *C. violaceum* CV12472 produces violacein when growing normally, the ability of the fractions to inhibit violacein in *C. violaceum* CV12472 indicates the inhibition of signal molecule production. The mutant strain *C. violaceum* CV026 is unable to produce violacein without an external acyl homoserine lactone (AHL) being supplied to respond to this hormone in a signal-reception manner. The fractions inhibited violacein production in *C. violaceum* CV026 when AHL was supplied, indicating that the fractions also inhibited signal reception. The QS inhibition zones are indicated by the cream-colored circles on the violet-colored lawn as shown in Figure 4. Bacteria act like unicellular organisms at low densities but at high densities, they behave like multicellular organisms through quorum sensing, involving communication through signal molecules and autoinducers, which coordinates their behavior and makes them become virulent [66]. The results indicate that the fractions are able to disrupt many virulent processes in bacteria through a QS mechanism. Swarming motility is regulated by QS and helps bacteria move towards nutrient-rich zones and also to colonize surfaces before preparing to establish biofilms, meaning that the inhibition of swarming movements can also avoid the incidence of bacterial spread and contamination in the environment [67]. The swarm fronts at different concentrations of *S. minor* fractions are indicated in Figure 4. Therefore, it is beneficial to observe that *S. minor* extracts inhibit swarming movements in pathogenic bacteria.

*S. minor* has shown good potential in inhibiting bacterial growth. This may be attributed to the phenolic compounds since the polar extracts rich in phenolic compounds displayed highest activity, among which the methanol extract was more active than the DCM extract [51]. In this aforementioned study, the minimum inhibitory concentration (MIC) ranged from 0.1 to 3.13 mg/mL for the most effective polar extract while in our study, MIC values ranged from 0.3125 to 2.5 mg/mL for the most effective polar extract (AQE). This indicates that *S. minor* extracts can be used to develop natural antimicrobials. Substances inhibiting the growth of microbial cells or killing them are referred to as antimicrobials and are used in medicine to overcome infections [68]. Bacteria and fungi cause many foodborne diseases, and many food safety measures are being envisaged to ensure the health of consumers; one of these involves foods with antimicrobial and nutritional properties [69]. Since *S. minor* is an edible plant, its safety and nutritional potential are guaranteed while its antimicrobial potential has been reported here. Our study shows that the fractions of *S. minor* were able to inhibit bacterial and fungal growth, possibly due to the contribution of the phenolic compounds. *S. minor* exhibited antimicrobial activity on a range of bacteria and fungi, which was related to its phenolic content and compounds [54]. However, when antimicrobials are poorly used, microbial resistance can occur, and microbial resistance is very dangerous for food contamination and human health. Despite the fact that *S. minor* has shown good antimicrobial effects in many studies [34,61], the antibiofilm effects of this plant are still under-studied. Microbial resistance can arise from biofilm formation and other quorum-sensing mediated processes, and hence, inhibiting biofilms and QS are suitable means of preventing microbial resistance, drug efflux pump activation, virulence and harm towards the host [70,71]. All the fractions inhibited biofilm formation in a concentration-dependent manner against *S. aureus* and *E. coli*. However, only the AQE inhibited biofilm formation in *C. albicans.* Biofilm mediates antimicrobial resistance. Microbial biofilms are surface-attached colonies encapsulated inside an extracellular protective matrix, which makes the bacteria cells less susceptible to the effects of antibiotics than the non-adherent, planktonic cells [72]. Infections associated with biofilms are very difficult to cure compared to non-biofilm attacks. Infections are, as a result, extremely difficult to cure. The ability of the *S. minor* fractions to reduce microbial biofilm formation at MIC and sub-MIC concentrations indicates the potential of this food plant to prevent food contamination and the emergence of biofilm resistant pathogens. The fractions equally inhibited the swarming movement, which is an important step that occurs prior to biofilm formation in bacteria.

## 4. Materials and Methods

### 4.1. Plant Material and Extraction

The plant was collected from Tebessa area (35°24′14.5″ N 8°05′31.6″ E) in 2021 during the month of September 2021 and identified by Soraya Hioun of the Department of Natural and Life Sciences (living things and CJB/African Plant Database) of Echahid Cheikh Larbi University, Tebessa, Algeria. Voucher specimens were deposited under herbarium numbers CUP/JP/5-6 for *Sanguisorba minor* L. in the herbarium of the Biology Department at Echahid Cheikh Larbi Tebessi University, Tebessa. The voucher specimens were also compared with authentic vouchers as well as other established phenotypic aspects of *S. minor* as described previously [10,36]. The selected material was harvested and then dried in the shade before the study. The plant powder (700 g) was macerated with methanol/water (70/30 *v*/*v*) for 24 h, after which the mixture was filtered, the filtrate dried using a rotary evaporator (Buchi R-215 Rotavapor, Flawil, Switzerland) under high pressure, and the methanolic extract was obtained. The crude extract was dissolved in hot distilled water, subjected to liquid–liquid extraction with different solvents and then filtered. The obtained filtrate was extracted using dichloromethane, ethyl acetate, and n-butanol with increasing solvent polarity to give the DCM, AcOEt, and BuOH fractions, respectively. The residue was equally dried and consisted of the aqueous fraction.

### 4.2. Total Phenolic and Total Flavonoid Content

The total phenolic content (TPC) was quantified using the Folin–Ciocalteu (F-C) method with little modification [73,74]. Briefly, 1000 μL of each sample and 1000 μL of F-C reagent were mixed and left for 2 min, and then 800 μL of 7.5% (*w*/*v*) sodium carbonate solution was added. The mixture was incubated at room temperature for 30 min in the dark. The absorbance of the obtained solutions was measured in a spectrophotometry apparatus at λ = 760 nm. Gallic acid concentrations ranging from 25 to 300 μg/mL were prepared, and a calibration curve was obtained using a linear fit. The samples were analyzed in triplicate to ensure the reproducibility of the results. The total flavonoid content (TFC) was determined by the aluminum chloride method with slight modification [13,75], where 300 μL of AlCl_3_ (1:10 *w*/*v*) was added to 0.5 mL of each sample, mixed, and left to react at room temperature for 10 min, to measure after that their absorbance at 510 nm. Catechin concentrations ranging from 0 to 1200 μg/mL were prepared, and a standard calibration curve was obtained using a linear fit.

### 4.3. Determination of Total Condensed Tannins

This assay was performed following the vanillin method with HCl. The method is based on the formation of red complexes which are formed by the reaction of vanillin with the terminal flavonoid group [76,77]. This is explained by the reaction of tannins which transform into red-colored anthocyanins [78]. A volume of 50 μL of each extract was added to 1500 μL of the vanillin/methanol solution at 4%, and then mixed vigorously; a volume of 750 μL of concentrated hydrochloric acid (HCl) was added. The mixture obtained was left at ambient temperature for 20 min. Absorbance was measured at 550 nm against a blank. Different concentrations between 0 and 1000 µg/mL prepared from a stock solution of catechin allowed the calibration curve to be plotted.

### 4.4. Determination of Total Anthocyanin by Absorption Spectroscopy

This is based on the reversible structural transformation of anthocyanin at different pH values [79] and the function of the change in color (color at pH = 1.0 and colorless at pH = 4.5). The advantage of the pH-differential method is that it allows a precise and rapid measurement of the total anthocyanin. The sample was diluted using (a) 0.025 M potassium chloride solution at pH 1.0, and (b) 0.4 M sodium acetate solution at pH 4.5. The blank consists of 100 μL of methanol acidified by HCl 0.1% and 4.9 mL of buffer solution (pH = 1.0 and pH = 4.5). The absorbance was measured at 520 nm and 700 nm, then the absorbance A calculated as follows:

$$A=\left(A_{520}-A_{700}\right)pH-\left(A_{520}-A_{700}\right)pH$$

### 4.5. HPLC–DAD Phenolic Profiles of Plant Extracts

The phenolic compounds in the acacia extracts were detected and quantified using reversed-phase high-performance liquid chromatography (RP-HPLC) coupled with a diode array detector (DAD) as described previously [80,81]. Briefly, known weights of each extract were dissolved in water/methanol (80/20) and then filtered on a sterile 0.20 μm disposable filter disk for liquid chromatography, and an Intersil ODS-3 reverse phase C18 column was used for the separation employing a 1.0 mL/min solvent flow rate and 20 μL injection volume. Two mobile phases: A (0.5% acetic acid H_2_O) and B (0.5% acetic acid in CH_3_OH). A gradient elution was applied as follows: 0–10% B (0–0.01 min); 10–20% B (0.01–5 min); 20–30% B (5–15 min); 30–50% B (15–25 min); 50–65% B (25–30 min); 65–75% B (30–40 min); 75–90% B (40–50 min) 90–10% B (50–55 min). A photodiode array detector set at 280 nm wavelength was employed in the detection, and the UV data together with retention times were compared with authentic standards. Each analysis was performed three times. A calibration plot established through the elution of known concentrations (0.0, 0.00782, 0.01563, 0.03125, 0.0625, 0.125, 0.25, 0.5, and 1.0 ppm) of authentic compounds was used for the identification and quantification of the plant constituent phenolic compounds. Twenty-six phenolic standards (gallic, *p*-hydroxy benzoic, protocatechuic, ellagic, chlorogenic, *trans*-cinnamic, 3-hydroxy benzoic, vanillic, syringic, *p*-coumaric, rosmarinic and ferulic acids; catechin, kaempferol, hesperetin, pyrocatechol vanillin, 6,7-dihydroxy coumarin, coumarin, rutin, myricetin, chrysin, luteolin, apigenin taxifolin, and quercetin) were used. The results were expressed as µg per g dry weight of the extract.

### 4.6. Evaluation of Antioxidant Activity

Lipid peroxidation inhibition measurement activity was performed by the β-carotene-linoleic acid test system with slight modifications [82]. The DPPH^•^ and ABTS^•+^ radical scavenging activities were evaluated spectrophotometrically as previously described [83,84]. The cupric-reducing antioxidant capacity (CUPRAC) test was performed using the method reported earlier [85,86]. BHA (butylated hydroxyanisole) and α-tocopherol were used as antioxidant standards for comparison of the β-carotene-linoleic acid, DPPH^•^, ABTS^•+^, and CUPRAC assays. The metal chelating activity of the extracts for Fe^2+^ was tested spectrophotometrically [87]. EDTA was the reference compound. Depletion of H_2_O_2_ antioxidant assay was performed as described elsewhere [88]. Antioxidant activity results are given as 50% inhibition concentration (IC_50_).

### 4.7. Anticholinesterase Activity

Anticholinesterase activity was measured spectrophotometrically by determining acetylcholinesterase (AChE) and butyrylcholinesterase (BChE) enzyme inhibitions as described by Ellman with minor modifications [89,90]. Electric eel AChE and horse serum BChE were used, while acetylthiocholine iodide and butyrylthiocholine chloride were employed as reaction substrates. The activity of the cholinesterase was monitored using DTNB (5,5′-Dithio-bis(2-nitrobenzoic) acid). Galantamine was used as a reference compound.

### 4.8. In Vitro Antidiabetic (α-Amylase and α-Glucosidase Inhibition) Assay

The α-amylase inhibitor activity was evaluated by using starch-iodine method with some modifications [15]. The enzyme α-amylase from porcine pancreas was used, and enzyme solution was prepared with phosphate buffer (20 mM pH = 6.9 phosphate buffer prepared with 6.0 mM NaCl). Then, 50 µL of α-amylase and 25 µL of sample solutions were mixed in a 96-well microplate. The mixture was pre-incubated for 10 min at 37 °C. Then, 50 µL of starch solution (0.05%) was added and incubated for 10 min at 37 °C. Following incubation, the reaction was completed by adding HCl (0.1 M, 25 µL) and Lugol (100 µL) solutions, and the absorbance was recorded at 565 nm.

The α-glucosidase inhibitor activity was evaluated according to the method described previously [91]. A measure of 50 µL of phosphate buffer (0.01 M pH 6.9), 10 µL of sample solution, 50 µL of α-glucosidase from *Saccharomyces cerevisiae* in phosphate buffer (0.01 M pH 6.0) and 25 µL of PNPG (4-N-nitrophenyl-α-D-glucopyranoside) in phosphate buffer (0.01 M pH 6.9) were mixed in a 96-well microplate. Then, the solution was incubated for 20 min at 37 °C. Acarbose was used as standard compound for both analyses. Results are given as percentage inhibition (%) at 200 µg/mL which was the highest test concentration.

### 4.9. Microbial Strains

The microorganisms *Staphylococcus aureus* ATCC 25923, *Escherichia coli* ATCC 25922 and *Candida albicans* ATCC 10239 were used for antimicrobial and antibiofilm studies. *Pseudomonas aeruginosa* PA01 was used in the swarming inhibition, while *Chromobacterium violaceum* CV12472 and *Chromobacterium violaceum* CV026 were used for violacein and quorum-sensing inhibitions, respectively.

### 4.10. Determination of Minimum Inhibitory Concentrations

Minimal inhibitory concentration (MIC) was determined by the broth dilution method described by the Clinical and Laboratory Standards Institute [92]. The MIC is the lowest extract concentration that yields no visible microbial growth. The test medium was Mueller–Hinton broth, and the density of bacteria was 5 × 10^5^ colony-forming units (CFU)/mL. Cell suspensions (100 μL) were inoculated into the wells of 96-well microtiter plates in the presence of extracts with different final concentrations (5, 2.5, 1.25, 0.625, 0.3125, 0.1562, 0.0781 mg/mL). The inoculated microplates were incubated at 37 °C for 24 h before being read.

### 4.11. Effect of Extracts on Bacterial Biofilm Formation

The ability of *S. minor* extracts at MIC and sub-MIC concentrations to inhibit biofilm by test microorganisms was evaluated with a microplate biofilm assay [93]. Briefly, 1% of overnight grown cultures of isolates were added to 200 μL of fresh Tryptose-Soy Broth (TSB) supplemented with 0.25% glucose and cultivated in the presence and absence of extracts without agitation for 48 h at 37 °C. The wells containing TSB+ cells only served as control. After incubation, planktonic bacteria were removed by gently washing them with distilled water. The biofilm colonies were subsequently stained by filling wells with 200 μL of 0.1% crystal violet solution and then allowed to stand for 10 min at room temperature. Wells were rinsed with distilled water using a micro-pipette to remove the unabsorbed crystal violet. A measure of 200 μL of 33% glacial acetic acid (for Gram-positive bacteria) or ethanol 70% (for Gram-negative bacteria or fungi) was filled into the wells. After shaking, 125 μL was pipetted from each of the wells into a sterile tube, and the volume was adjusted to 1 mL using distilled water. Finally, the optical density (OD) of each well was measured at 550 nm (Thermo Scientific Multiskan FC, Vantaa, Finland). The percentage of inhibition of biofilm by the tested extracts was calculated using the formula:

$$Biofilminhibition(\%)=\frac{{OD550}_{Control}-{OD550}_{Sample}}{{OD550}_{Control}}$$

### 4.12. Bioassay for Quorum-Sensing Inhibition (QSI) Activity Using C. violaceum CV026

Inhibition of quorum sensing was determined as described elsewhere with little modification [94,95]. A measure of 5 mL of lukewarm molten Soft Top Agar (1.3 g agar, 2.0 g tryptone, 1.0 g sodium chloride, 200 mL deionized water) was seeded with 100 µL of an overnight grown culture of CV026, and 20 µL of 100 µg/mL acyl-homoserine lactone (AHL) was added as exogenous hormone source. It was mixed gently and poured carefully over the surface of a sterile solidified LBA plate as an overlay. Some 5 mm diameter wells were made on each plate after solidification of the overlay and each well was filled with 50 µL of MIC and sub-MIC concentrations of filter-sterilized *S. minor* extracts. Each experiment was carried out in triplicate and the plates were incubated in an upright position at 30 °C for 3 days, after which the diameters of the quorum-sensing inhibition zones were measured. A white or cream-colored halo around this well against a purple lawn of activated CV026 bacteria was an indication of QSI, and its diameter was measured in millimeters.

### 4.13. Violacein Inhibition Assay Using C. violaceum CV12472

*S. minor* extracts were subjected to qualitative analysis of QSI potential for their ability to inhibit violacein production by *C. violaceum* ATCC 12,472 [96]. Overnight grown cultures (10 µL) of *C. violaceum* (adjusted to 0.4 OD at 600 nm) were added to sterile microtiter plates containing 200 µL of Luria–Bertani (LB) broth and incubated in the presence and absence of MIC and sub-MICs of extracts. LB broth containing *C. violaceum* ATCC 12,472 was used as a positive control. These plates were incubated at 30 °C for 24 h and observed for the reduction in violacein pigment production. The absorbance was read at 585 nm. The percentage of violacein inhibition was calculated by following the formula:

$$Violacein\;inhibition\;\left(\%\right)=\frac{OD\;585\;control-OD585\;sample\;}{OD\;585\;control}\times 100$$

### 4.14. Swarming Motility Inhibition on Pseudomonas aeruginosa PA01

Swarming motility inhibition was evaluated according to a method previously described [97,98]. Briefly, overnight-grown cultures of *P. aeruginosa* PA01 strain were point inoculated in the center of swarming plates consisting of 1% peptone, 0.5% NaCl, 0.5% agar, and 0.5% of filter-sterilized D-glucose with various MIC and sub-MIC concentrations of *S. minor* extracts (MIC, MIC/2, and MIC/4) and the plate without the extract was maintained as control. Plates were incubated at an appropriate temperature in an upright position for 18 h. The swarming migration was recorded by following swarm fronts of the bacterial cells.

### 4.15. Statistical Analysis

Activity assays were performed in triplicate. The data were recorded as means ± standard error of the means (SEM). Minitab 16 statistical software was used to determine the significant differences between means using one-way ANOVA (analysis of variance), in which *p* < 0.05 was regarded as significant.

## 5. Conclusions

Food plants can provide the medicinal as well as the nutritional benefits that are necessary for the proper functioning of the body. The young leaves of *Sanguisorba minor* are edible and are also used in mixed salads, for flavoring drinks and improving the taste of wines and beverages. This plant also possesses important biological properties which can improve human health by preventing metabolic diseases, amongst other illnesses. HPLC-DAD analyses of *S. minor* fractions indicated that the plant is rich in phenolic compounds. One of the important classes of compounds in foods and medicine includes phenolic compounds. The fractions of *S. minor* exhibited good antioxidant activity, indicating that they can prevent oxidative stress-related ailments. The extracts inhibited cholinesterases and carbohydrate digestive enzymes, suggesting a reduction in Alzheimer’s disease and diabetes. Biofilm formation, as well as other quorum-sensing processes were disrupted by the fractions, suggesting that *S. minor* could be a source of new antibiotic foods, which could reduce pathogenic virulence and resistance in bacteria. The results indicate a great potential of *S. minor* as a food preservative and additive that could help to increase the shelf life of other food substances.

## Figures and Tables

**Table 1:** Total polyphenol content (TPC), flavonoid content (FC), tannin content (TC), and anthocyanin content (AC) contained in *S. minor* fractions.

Sample	TPC mg GAE/g DW ^(a)^	FC mg QE/g DW ^(b)^	TC mg CE/g DW ^(c)^	AC mg CGE/g DW ^(d)^
**DCME**	240.0 ± 4.9	155.7 ± 1.1	113.5 ± 3.0	6.5 ± 0.7
**EAE**	811.0 ± 4.7	254.9 ± 3.5	129.7 ± 2.1	2.7 ± 0.1
**BE**	461.0 ± 4.8	101.6 ± 2.0	102.2 ± 4.3	8.6 ± 0.2
**AQE**	232.0 ± 4.4	78.4 ± 2.2	58.6 ± 1.6	1.5 ± 0.0

(^a^) Expressed in Gallic acid equivalent (GAE); (^b^) expressed in Quercetin equivalent (CE); (^c^) expressed in Catechin equivalent (CE); (^d^) expressed in Cyanidin-3-galactoside equivalent (CGE).

**Table 2:** Phenolic composition of the *S. minor* fractions by HPLC-DAD (µg/g) ^a^.

No.	Phenolic Compounds	RT (min)	DCME	EAE	BE	AQE	Reference
**1**	Gallic acid	5.70	-	-	-	-	
**2**	Protocatechuic acid	8.85	0.85 ± 0.06	10.20 ± 0.17	-	-	[1,11,12,23]
**3**	Catechin	10.68	-	-	-	-	
**4**	Pyrocatechol	11.04	0.52 ± 0.04	13.44 ± 0.23	-	-	[1,12]
**5**	Chlorogenic acid	12.35	-	-	-	-	
**6**	*p*-Hydroxy benzoic acid	12.77	-	22.63 ± 0.28	2.52 ± 0.15	8.15 ± 0.08	[1,11]
**7**	6.7-Dihydroxy coumarin	14.10	-	-	-	-	
**8**	Caffeic acid	15.09	5.05 ± 0.12	70.49 ± 0.35	-	1.86 ± 0.05	[12,23]
**9**	3-Hydroxy benzoic acid	15.98	-	-	-	-	
**10**	Syringic acid	16.56	0.41 ± 0.02	75.82 ± 0.48	-	2.97 ± 0.10	[1,11,23]
**11**	Vanillin	17.78	5.68 ± 0.10	2.85 ± 0.08	-	2.70 ± 0.08	[1,23]
**12**	*p*-Coumaric acid	20.56	-	120.1 ± 0.75	-	10.44 ± 0.12	[1,12]
**13**	Taxifolin	21.26	-	-	-	-	
**14**	Ferulic acid	22.14	-	25.34 ± 0.25	0.85 ± 0.04	0.57 ± 0.05	[1,11,12]
**15**	Coumarin	24.49	-	-	-	-	
**16**	Rutin	25.30	-	85.47 ± 0.44	278.4 ± 1.20	32.87 ± 0.23	[1,11,12]
**17**	Ellagic acid	26.11	-	-	-	-	
**18**	Rosmarinic acid	26.57	10.22 ± 0.17	124.5 ± 0.80	0.98 ± 0.06	-	[1,12,14]
**19**	Myricetin	27.35	-	-	-	-	
**20**	Quercetin	30.83	7.19 ± 0.21	9.73 ± 0.35	-	-	[1,23]
**21**	*trans*-Cinnamic acid	31.33	-	-	-	-	
**22**	Luteolin	31.70	3.42 ± 0.25	133.6 ± 0.70	-	-	[1,11,14]
**23**	Hesperetin	32.14	-	-	-	-	
**24**	Kaempferol	33.21	-	-	-	-	
**25**	Apigenin	33.77	84.75 ± 0.60	156.8 ± 0.95	-	-	[1,12,23]
**26**	Chrysin	38.40	-	-	-	-	

^a^ Values expressed are means ± S.E.M. of three parallel measurements (*p* < 0.05). -: not detected.

**Table 3:** Antioxidant activity of the *S. minor* fractions by different assays ^a^.

Sample	β-Carotene Assay	DPPH^•^ Assay	ABTS^•+^ Assay	CUPRAC Assay	Metal Chelating Assay	H_2_O_2_ Assay
	IC_50_ (µg/mL)	IC_50_ (µg/mL)	IC_50_ (µg/mL)	A_0.50_ (µg/mL)	IC_50_ (µg/mL)	IC_50_ (µg/mL)
**DCME**	51.30 ± 0.98	7.16 ± 0.80	62.50 ± 0.94	8.36 ± 0.25	5.83 ± 1.20	53.60 ± 0.36
**EAE**	7.44 ± 0.28	17.37 ± 0.45	9.27 ± 0.33	21.70 ± 0.51	19.48 ± 0.88	49.20 ± 0.21
**BE**	20.86 ± 0.84	39.51 ± 0.96	28.19 ± 0.57	34.81 ± 0.65	51.65 ± 1.14	66.60 ± 0.39
**AQE**	92.48 ± 0.76	128.30 ± 1.30	87.40 ± 1.12	83.53 ± 0.55	102.90 ± 0.98	71.00 ± 0.82
**α-Tocopherol**	2.10 ± 0.05	38.20 ± 0.50	35.50 ± 0.55	60.20 ± 0.45	NT	NT
**BHA**	1.50 ± 0.03	19.50 ± 0.30	12.70 ± 0.10	25.40 ± 0.38	NT	NT
**EDTA**	NT	NT	NT	NT	5.50 ± 0.35	NT
**Ascorbic acid**	NT	NT	NT	NT	NT	28.70 ± 0.36

^a^ Values represent the means ± SEM of three parallel sample measurements (*p* < 0.05). NT: not tested.

**Table 4:** Anticholinesterase and antidiabetic activities of *S. minor* fractions.

	Cholinesterase Inhibitory Activity		Antidiabetic Activity	
	AChE	BChE	α-Glucosidase	α-Amylase
Sample	Inhibition (%) (at 200 µg/mL)			
**DCME**	32.75 ± 0.48	41.25 ± 0.69	55.18 ± 0.26	32.45 ± 0.43
**EAE**	36.11 ± 0.51	54.50 ± 0.74	41.09 ± 0.85	29.87 ± 0.67
**BE**	18.71 ± 0.45	24.80 ± 0.61	32.73 ± 0.90	25.51 ± 0.82
**AQE**	10.42 ± 0.33	21.60 ± 0.78	21.44 ± 0.72	14.08 ± 0.26
**Galantamine**	88.70 ± 0.50	80.20 ± 0.30	NT	NT
**Acarbose**	NT	NT	86.51 ± 0.45	81.33 ± 0.90

Values represent the means ± SEM of three parallel sample measurements (*p* < 0.05). NT: not tested.

**Table 5:** Inhibition of violacein production in *C. violaceum* CV12472 by *S. minor* fractions.

Sample	MIC (mg/mL)	Violacein Inhibition (%)					
		MIC	MIC/2	MIC/4	MIC/8	MIC/16	MIC/32
**DCME**	0.625	100 ± 0.0	78.5 ± 1.9	49.4 ± 1.5	31.0 ± 0.7	16.1 ± 0.2	-
**EAE**	0.625	100 ± 0.0	61.9 ± 0.6	29.9 ± 1.0	13.0 ± 0.5	-	-
**BE**	2.5	100 ± 0.0	40.7 ± 0.8	19.8 ± 0.4	-	-	-
**AQE**	1.25	100 ± 0.0	61.9 ± 1.3	61.9 ± 1.1	24.9 ± 0.5	11.7 ± 0.3	-

-: no inhibition.

**Table 6:** Quorum-sensing inhibition zones in *C. violaceum* CV026 by *S. minor* fractions.

	Antiquorum-Sensing Inhibition Zones (mm)				
Sample	MIC (mg/mL)	MIC	MIC/2	MIC/4	MIC/8
**DCME**	1.25	14.0 ± 1.5	10.0 ± 1.0	-	-
**EAE**	1.25	13.0 ± 0.8	09.5 ± 0.2	-	-
**BE**	0.625	14.5 ± 0.6	10.0 ± 0.1	-	-
**AQE**	0.625	17.0 ± 1.2	15.0 ± 0.9	11.5 ± 0.4	09.0 ± 0.5

-: no inhibition.

**Table 7:** Swarming motility inhibition on *P. aeruginosa* PA01 by *S. minor* fractions.

Sample	100 µg/mL	75 µg/mL	50 µg/mL
**DCME**	18.4 ± 0.6	05.5 ± 0.2	-
**EAE**	34.9 ± 1.5	16.3 ± 0.7	06.2 ± 0.1
**BE**	39.5 ± 0.3	23.0 ± 0.6	10.5 ± 0.3
**AQE**	20.1 ± 0.5	03.8 ± 0.1	-

-: no inhibition.

**Table 8:** Antimicrobial (MIC) and antibiofilm activities of *S. minor* fractions.

Microorganisms		Sample Codes			
		DCME	EAE	BE	AQE
		MIC (mg/mL)			
*S. aureus*		1.25	1.25	0.625	0.312
*E. coli*		1.25	2.5	2.5	1.25
*C. albicans*		0.625	1.25	0.625	0.625
		**Biofilm inhibition (%)**			
*S. aureus*	MIC	71.25 ± 1.78	55.65 ± 0.59	76.14 ± 1.95	26.36 ± 0.27
	MIC/2	42.38 ± 0.96	23.82 ± 0.35	45.24 ± 0.85	8.29 ± 0.06
	MIC/4	16.92 ± 0.56	5.87 ± 0.18	27.32 ± 0.66	-
	MIC/8	-	-	10.22 ± 0.15	-
*E. coli*	MIC	60.16 ± 1.24	56.65 ± 1.05	46.13 ± 0.78	51.11 ± 0.64
	MIC/2	26.63 ± 0.76	30.14 ± 0.65	21.34 ± 0.25	29.21 ± 0.44
	MIC/4	13.15 ± 0.28	18.62 ± 0.44	6.18 ± 0.10	17.33 ± 0.15
	MIC/8	-	06.21 ± 0.32	-	05.27 ± 0.1
*C. albicans*	MIC	-	-	-	11.26 ± 0.13
	MIC/2	-	-	-	02.44 ± 0.05
	MIC/4	-	-	-	-

-: no inhibition.

## Data Availability

The data supporting the reported results can be obtained from the corresponding author upon reasonable request.
